# Supplementary material for: Fibroblasts Promote Resistance to KRAS Silencing in Colorectal Cancer Cells
Source: Cancers (Basel). 2024 Jul 20;16(14):2595. doi: 10.3390/cancers16142595 (PMC11274566; doi:10.3390/cancers16142595)
Supplement: Supplementary file 1 [file cancers-16-02595-s001.zip › Supplementary Figure S2.pdf]

# = Fibroblast Activation for the Conditioned Media of Spheres =

12.11.2020

12% Polyacrylamide Gel | Run: 120 V; 3h | 20µL of each sample and 6 µL of marker

Primary antibodies:

- HSP70, Mouse, M(1:10.000); 70 kDA
- α-SMA, Mouse, M (1:250); 42 kDA
- GAPDH, Mouse, M (1:10 000); 37 kDa

* M: blocked in 5% Milk in PBST

Secondary antibodies:

- Anti-mouse, M (1:20.000);
- Anti-mouse, M (1:3000);
- Anti-mouse, M (1:20 000);

* M: blocked in 5% Milk in PBST

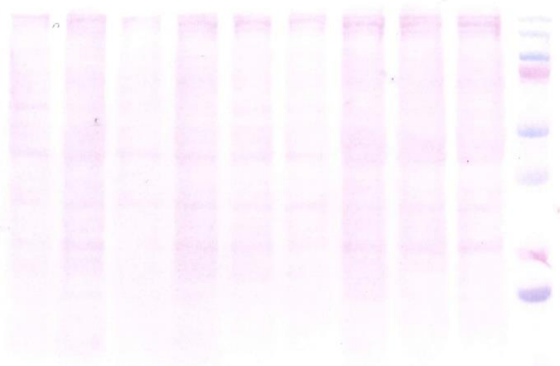

→ Ponceau 11.10.20

Experimental Conditions:

1. MC Esferas 29.10.2019 CDT (CTRL+)
2. MC Esferas 22.09.2020 CD (A)
3. MC Esferas 22.09.2020 CDT (A)
4. MC Esferas 29.09.2020 CD
5. MC Esferas 29.09.2020 CDT
6. MC Esferas 02.10.2020 CD
7. MC Esferas 02.10.2020 CDT
8. MC Esferas 06.10.2020 CD
9. MC Esferas 06.10.2020 CDT

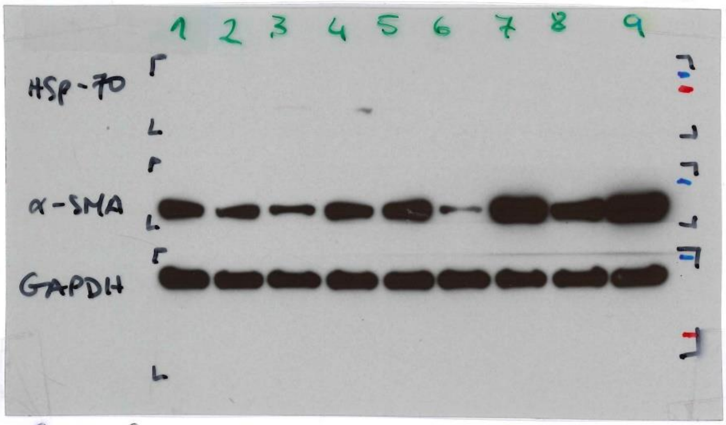

Exposure: 5 min, Machine

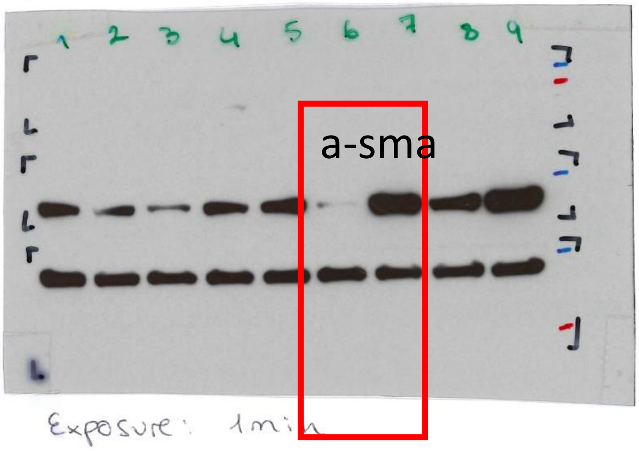

Exposure: 1 min

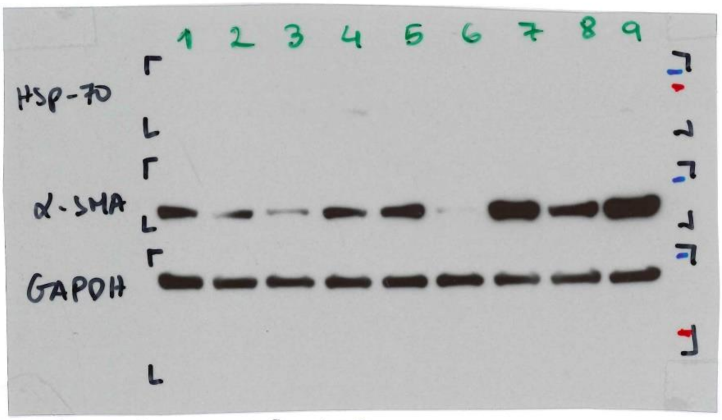

Exposure: 30 seg

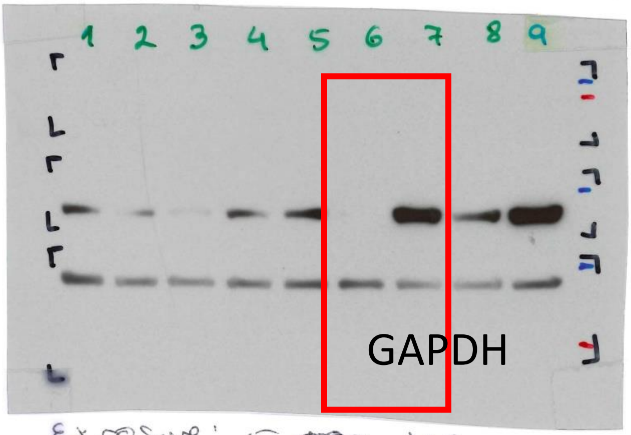

Exposure: 5 seg

Figure S1 a) a-sma

# = Confirmation of KRAS silencing in colonospheres =

10.11.2021

12% Polyacrylamide Gel | Run: 100 V; 3h | 20µL of each sample and 6 µL of marker

Primary antibodies:

- Hsc-70, Mouse, M[1:8000]; 70 kDA (mama) 05.09.19
- GAPDH, Mouse, M [1: 10.000]; 37 kDa 20.10.21
- KRAS, Mouse, M [1: 4000]; 21 kDa 20.10.21

* M: blocked in 5% Milk in PBST

Secondary antibodies:

- Anti-mouse, M [1: 8000]
- Anti-mouse, M [1: 16.000]
- Anti-mouse, M [1: 8000]

* M: blocked in 5% Milk in PBST

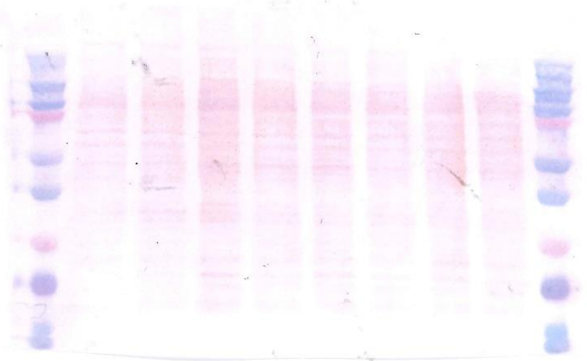

Experimental Conditions :

1. Marker
2. SW480 siNT 09.06.2021
3. SW480 siKRAS 09.06.2021
4. HCT15 siNT 17.06.2021
5. HCT15 siKRAS 17.06.2021
6. HCT15 siNT 01.07.2021
7. HCT15 siKRAS 01.07.2021
8. HCT116 siNT 20.08.2021
9. HCT116 siKRAS 20.08.2021
10. Marker

→ Ponceau

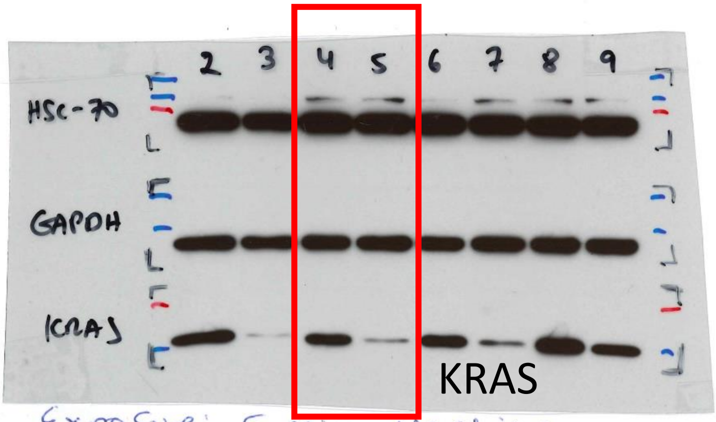

Exposure: 5 min. Machine

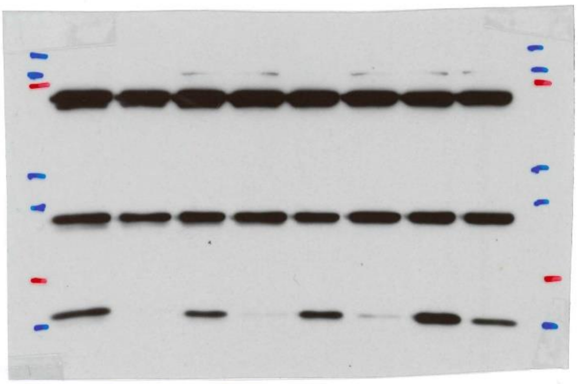

Exposure: 1 min

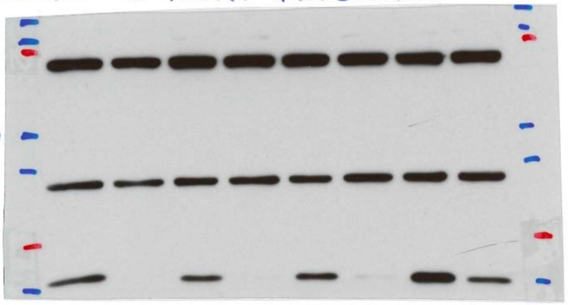

30 Ses

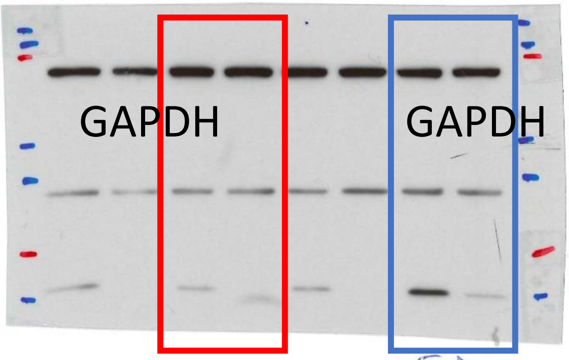

5 Ses

KRAS

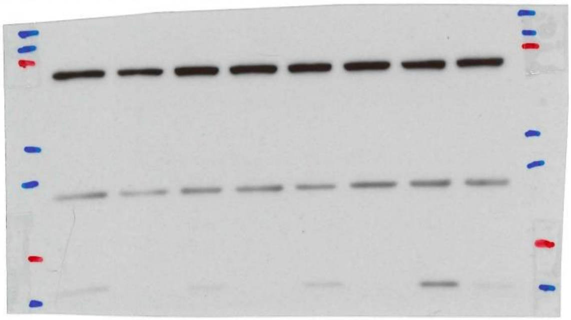

2 Ses

Figure S1. b) HCT15 48h

HCT116 48h

# = Confirmation of KRAS silencing in CRC cell lines =

20.01.2022

12% Polyacrylamide Gel | Run: 100 V; 3h | 20µL of each sample and 6 µL of marker

Primary antibodies:

- Hsc-70, Mouse, M[1:8000]; 70 kDA (mama) 05.09.19
- KRAS, Mouse, M [1: 4000]; 21 kDa 21.10.21
- GAPDH, Mouse, M [1: 10.000]; 37 kDa 20.10.21

* M: blocked in 5% Milk in PBST

Secondary antibodies:

- Anti-mouse, M [1: 8000]
- Anti-mouse, M [1: 8000]
- Anti-mouse, M [1: 16.000]

* M: blocked in 5% Milk in PBST

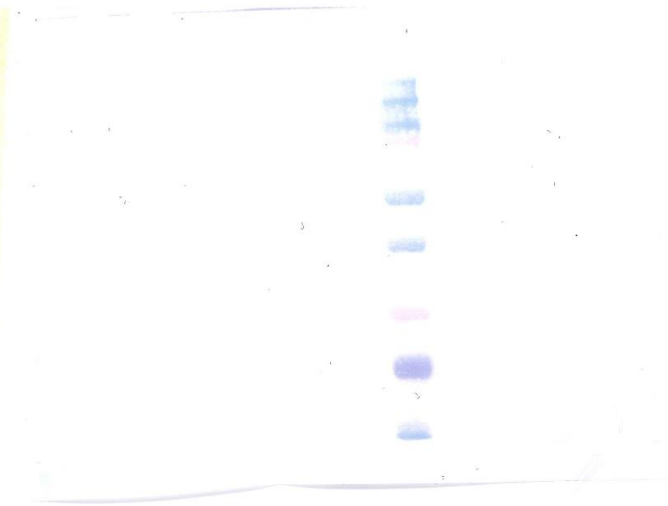

Experimental Conditions :

1. HCT15 siNT 08.02.2021
2. HCT15 siKRAS 08.02.2021
3. HCT116 siNT 03.09.2021
4. HCT116 siKRAS 03.09.2021
5. SW480 siNT 18.02.2021
6. SW480 siKRAS 18.02.2021
7. Marker
8. Empty
9. Empty
10. Empty

Figure S1. b) SW480 48h

ponceau

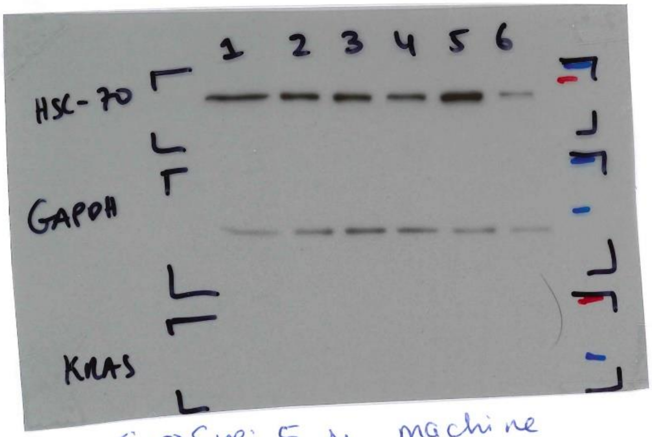

Exposure: 5 s machine

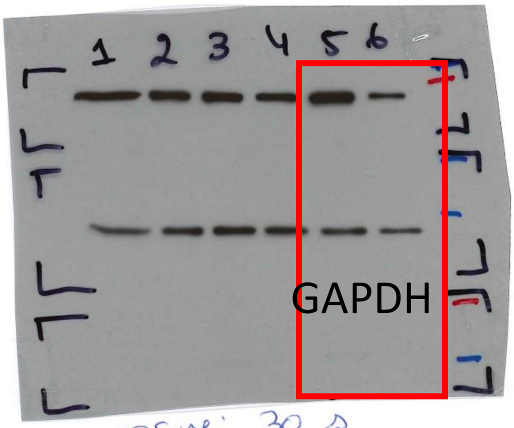

Exposure: 30 s

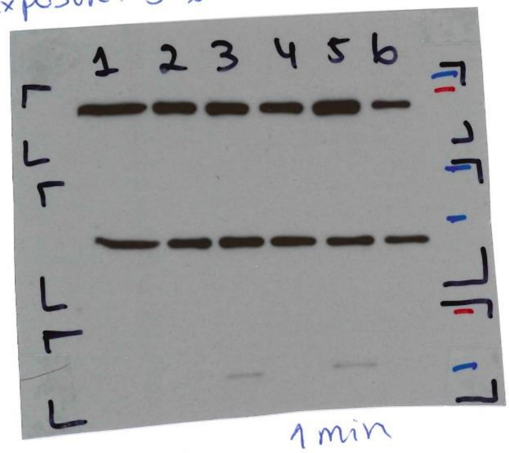

1 min

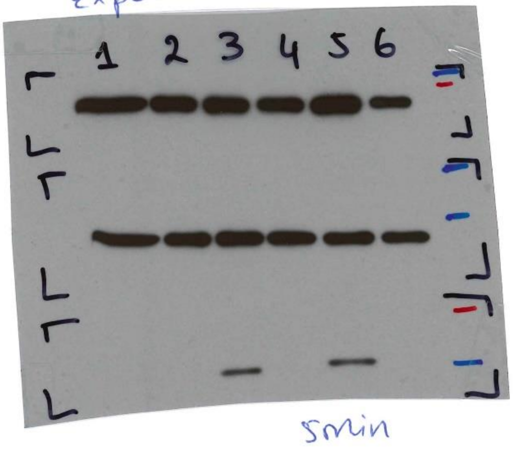

5min

Continuação do WB 20.07.2022

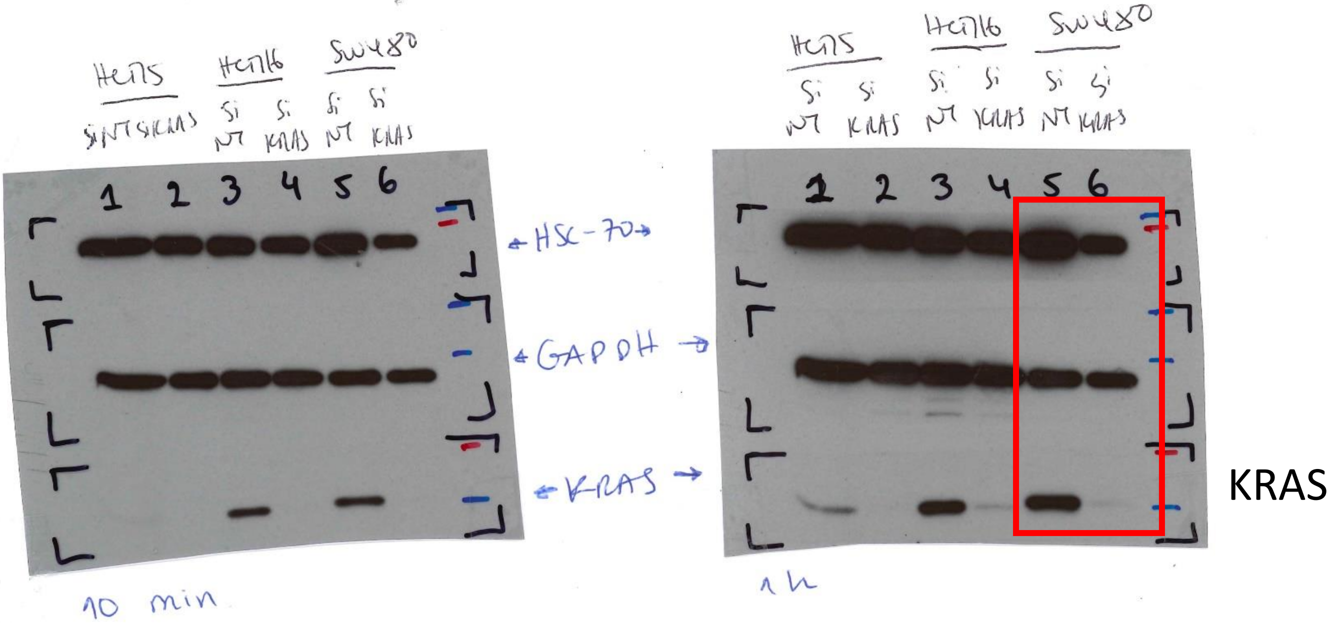

Figure S1. b) SW480 48h

# = Confirmation of KRAS silencing in Colonospheres=

24.04.2021

12% Polyacrylamide Gel | Run: 100 V; 3h | 20µL of each sample and 6 µL of marker

Primary antibodies:

- Hsp-70, Mouse, M[1:5000]; 70 kDA 17.03.21
- GAPDH, Mouse, M [1: 10.000]; 37 kDa 22.04.21
- KRAS (LSBIO), Mouse, M [1: 250]; 21 kDA 15.04.21

* M: blocked in 5% Milk in PBST

Secondary antibodies:

- Anti-mouse, M [1: 10.000]
- Anti-mouse, M [1: 20.000]
- Anti-mouse, M [1: 8000]

* M: blocked in 5% Milk in PBST

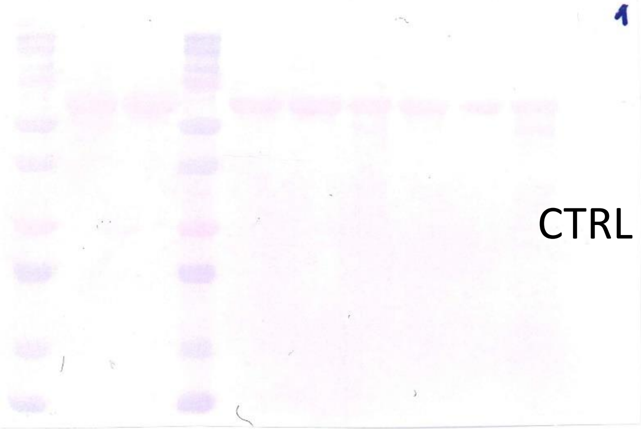

Experimental Conditions:

1. Marker
2. ESFERAS HCT15 siNT DMEM all sup 26.01.2021 } 15 µg
3. ESFERAS HCT15 siKRAS DMEM all sup 26.01.2021
4. Marker
5. ESFERAS HCT15 siNT DMEM all sup 13.02.2021 } 10 µg
6. ESFERAS HCT15 siNT DMEM TGF 13.02.2021
7. ESFERAS HCT15 siNT CCD18CO DMEM TGF 13.02.2021 — CTRL
8. ESFERAS HCT15 siKRAS DMEM all sup 13.02.2021
9. ESFERAS HCT15 siKRAS DMEM TGF 13.02.2021
10. ESFERAS HCT15 siKRAS CCD18CO DMEM TGF 13.02.2021

→ Ponceau

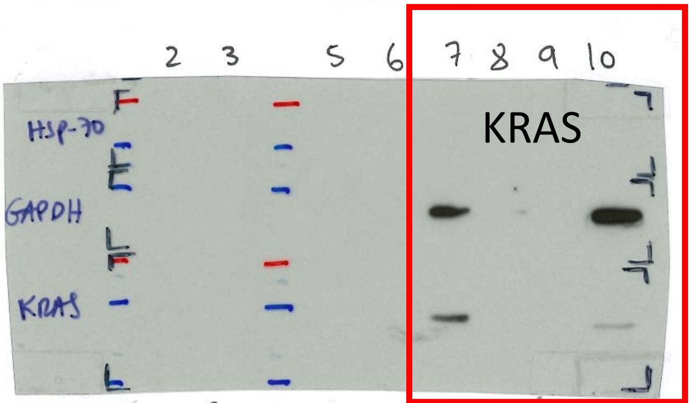

Exposure: 45 min. Machine

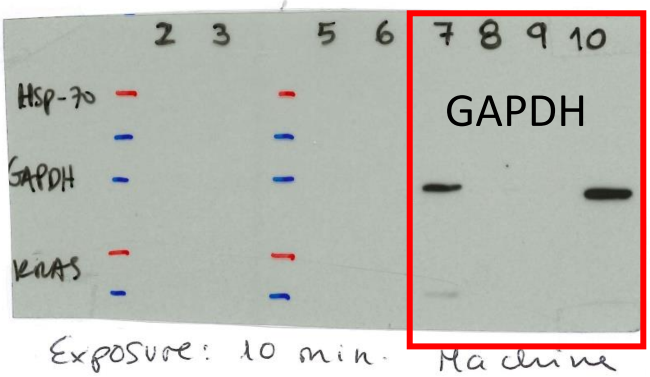

Exposure: 10 min. Machine

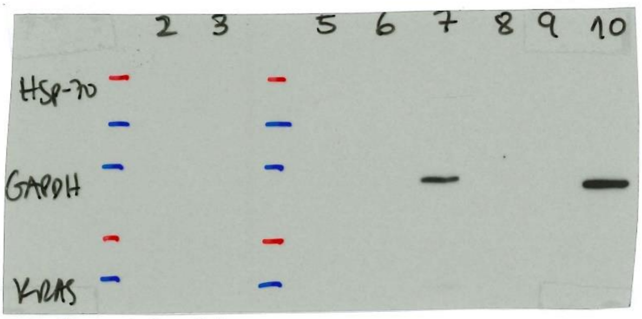

Exposure: 5 min

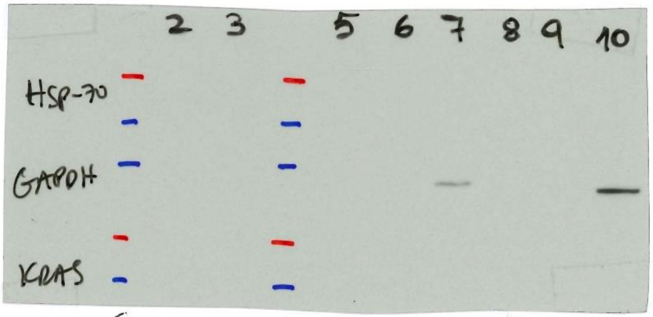

Exposure: 1 min

Figure S1. c) HCT15 after 5 days of sphere formation assay

# = Confirmation of KRAS silencing in Colonospheres=

24.04.2021

12% Polyacrylamide Gel | Run: 100 V; 3h | 20µL of each sample and 6 µL of marker

Primary antibodies:

- Hsp-70, Mouse, M[1:5000]; 70 kDA 17.03.21
- GAPDH, Mouse, M [1: 10.000]; 37 kDa 22.04.21
- KRAS (LSBIO), Mouse, M [1: 250]; 21 kDA 15.04.21

* M: blocked in 5% Milk in PBST

Secondary antibodies:

- Anti-mouse, M [1: 10.000]
- Anti-mouse, M [1: 20.000]
- Anti-mouse, M [1: 8000]

* M: blocked in 5% Milk in PBST

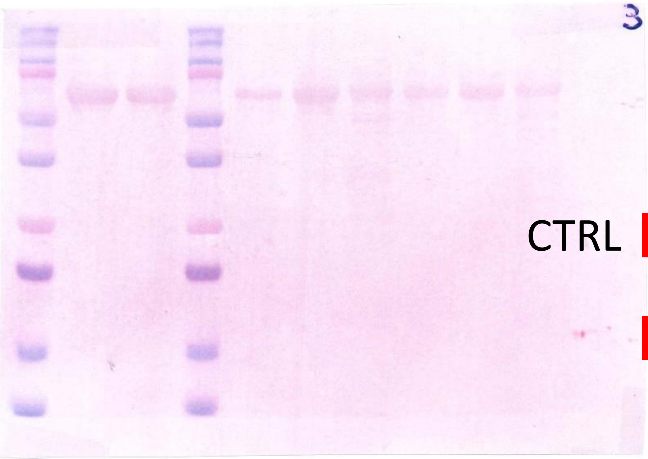

→ Ponceau

3 **Experimental Conditions:**

1. Marker
2. ESFERAS SW480 siNT DMEM all sup 06.02.2021 } 11 µg
3. ESFERAS SW480 siKRAS DMEM all sup 06.02.2021
4. Marker
5. ESFERAS HCT116 siNT DMEM all sup 21.02.2021 } 10 µg
6. ESFERAS HCT116 siNT DMEM TGF 21.02.2021
7. ESFERAS HCT116 siNT CCD18CO DMEM TGF 21.02.2021 (CTRL)
8. ESFERAS HCT116 siKRAS DMEM all sup 21.02.2021
9. ESFERAS HCT116 siKRAS DMEM TGF 21.02.2021
10. ESFERAS HCT116 siKRAS CCD18CO DMEM TGF 21.02.2021

Figure. S1. c) HCT116 after 5 days of sphere formation assay

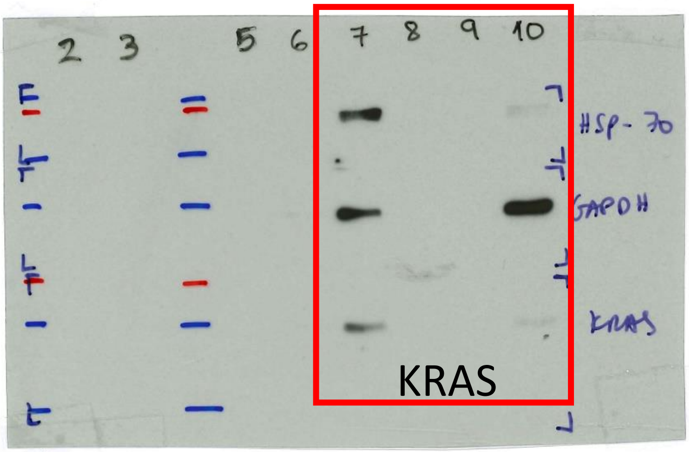

Exposure: 45 min. Machine

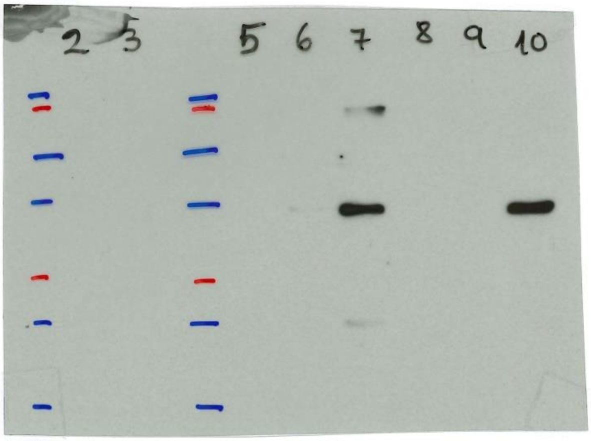

Exposure: 10 min

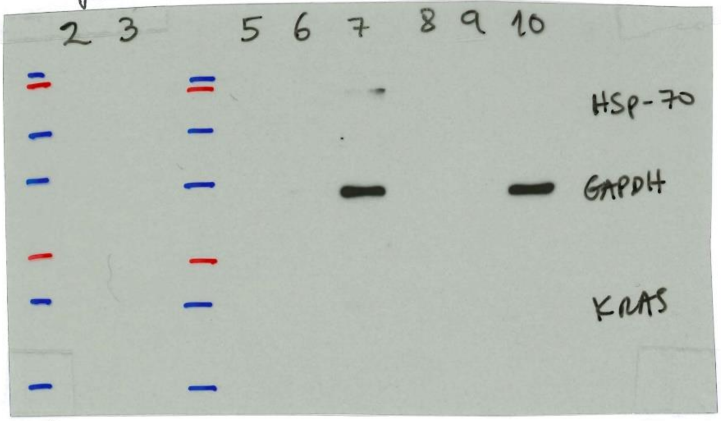

Exposure: 5 min

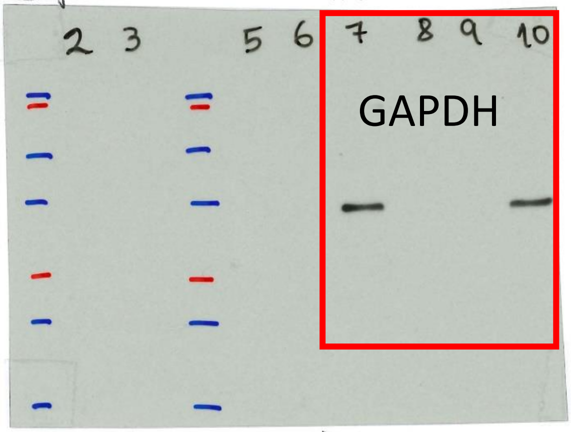

Exposure: 1 min

# = Confirmation of KRAS silencing in Colonospheres=

24.04.2021

12% Polyacrylamide Gel | Run: 100 V; 3h | 20µL of each sample and 6 µL of marker

Primary antibodies:

- Hsp-70, Mouse, M[1:5000]; 70 kDA 17.03.21
- GAPDH, Mouse, M [1: 10.000]; 37 kDa 22.04.21
- KRAS (LSBIO), Mouse, M [1: 250]; 21 kDA 15.04.21

* M: blocked in 5% Milk in PBST

Secondary antibodies:

- Anti-mouse, M [1: 10.000]
- Anti-mouse, M [1: 20.000]
- Anti-mouse, M [1: 8000]

* M: blocked in 5% Milk in PBST

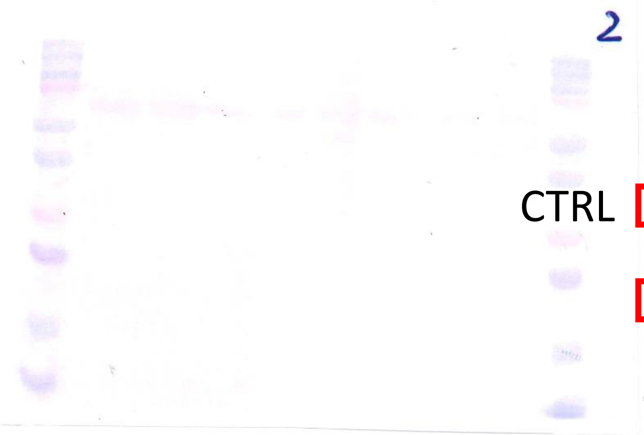

Experimental Conditions: 10 µs

1. Marker
2. ESFERAS HCT15 siNT DMEM all sup 02.02.2021
3. ESFERAS HCT15 siKRAS DMEM all sup 02.02.2021
4. ESFERAS SW480 siNT DMEM all sup 16.02.2021
5. ESFERAS SW480 siNT DMEM TGF 16.02.2021
6. ESFERAS SW480 siNT CCD18CO DMEM TGF 16.02.2021 — CTRL
7. ESFERAS SW480 siKRAS DMEM all sup 16.02.2021
8. ESFERAS SW480 siKRAS DMEM TGF 16.02.2021
9. ESFERAS SW480 siKRAS CCD18CO DMEM TGF 16.02.2021
10. Marker

→ Ponceau

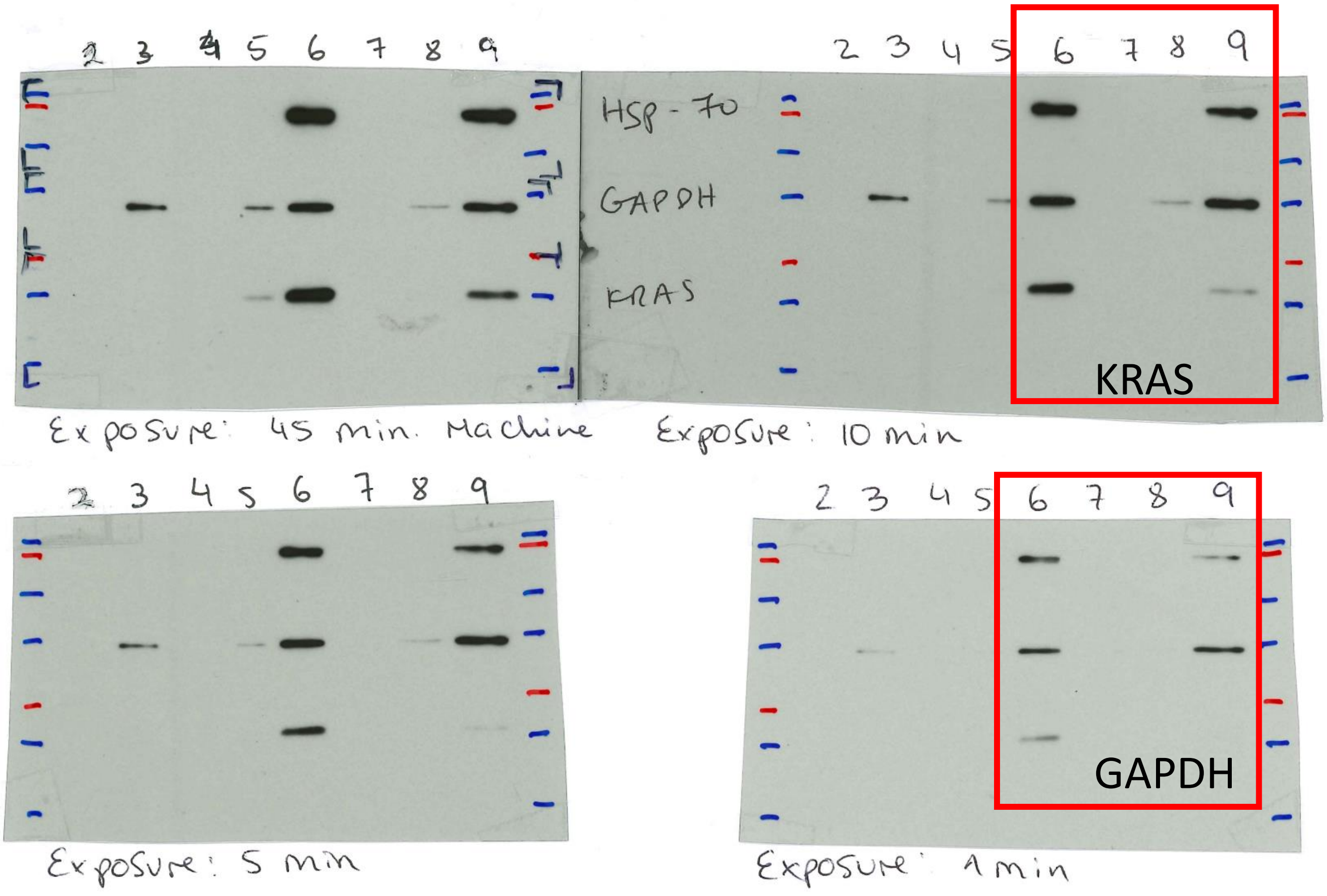

Figure S1. c) SW480 after 5 days of sphere formation assay

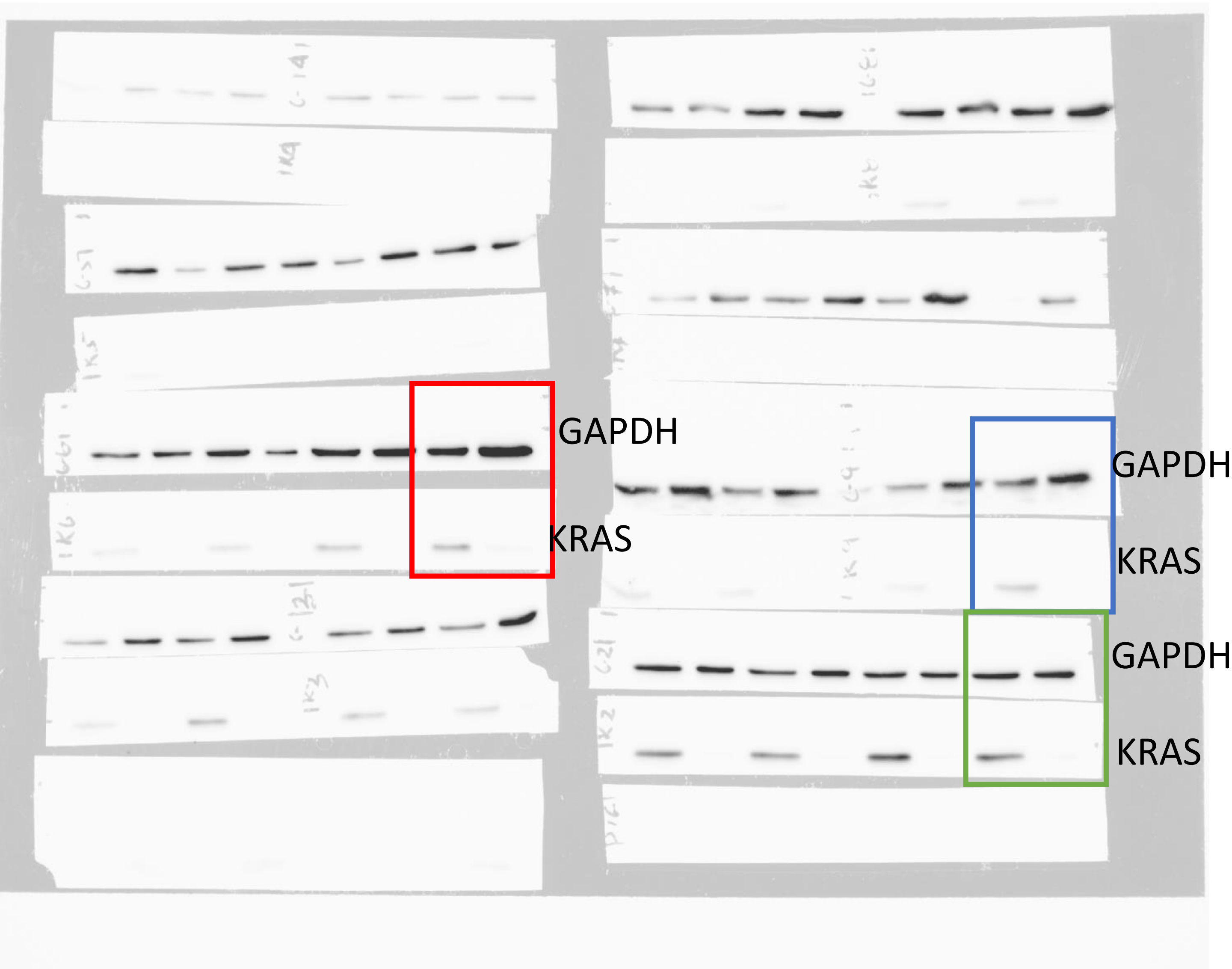

Figure S1. d) hct15 after 48h of silencing + treatment with CM
hct116 after 48h of silencing + treatment with CM
SW480 after 48h of silencing + treatment with CM
