# Supplementary figures and images for: Fibroblasts Promote Resistance to KRAS Silencing in Colorectal Cancer Cells
Source: Cancers (Basel). 2024 Jul 20;16(14):2595. doi: 10.3390/cancers16142595 (PMC11274566; doi:10.3390/cancers16142595)

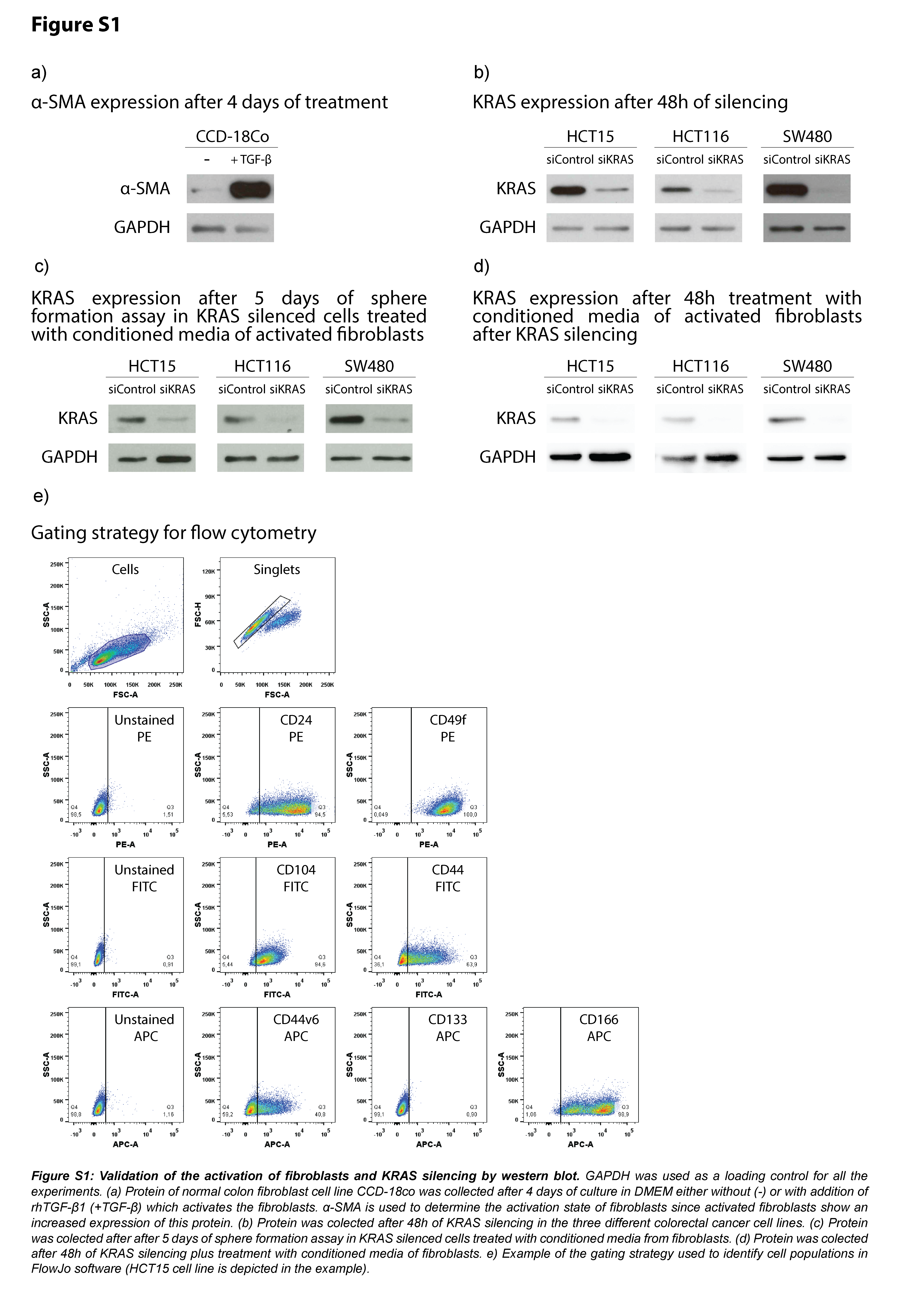

Supplement: Supplementary file 1 [file cancers-16-02595-s001.zip › Supplementary Figure S1.png]
